# A Novel Bio-Sensor Based on DNA Strand Displacement

**DOI:** 10.1371/journal.pone.0108856

**Published:** 2014-10-10

**Authors:** Xiaolong Shi, Zhiyu Wang, Chenyan Deng, Tao Song, Linqiang Pan, Zhihua Chen

**Affiliations:** Key Laboratory of Image Information Processing and Intelligent Control, School of Automation, Huazhong University of Science and Technology, Wuhan, Hubei, China; CNR, Italy

## Abstract

DNA strand displacement technology performs well in sensing and programming DNA segments. In this work, we construct DNA molecular systems based on DNA strand displacement performing computation of logic gates. Specifically, a class of so-called “DNA neurons” are achieved, in which a “smart” way inspired by biological neurons encoding information is developed to encode and deliver information using DNA molecules. The “DNA neuron” is bistable, that is, it can sense DNA molecules as input signals, and release “negative” or “positive” signals DNA molecules. We design intelligent DNA molecular systems that are constructed by cascading some particularly organized “DNA neurons”, which could perform logic computation, including AND, OR, XOR logic gates, automatically. Both simulation results using visual DSD (DNA strand displacement) software and experimental results are obtained, which shows that the proposed systems can detect DNA signals with high sensitivity and accretion; moreover, the systems can process input signals automatically with complex nonlinear logic. The method proposed in this work may provide a new way to construct a sensitive molecular signal detection system with neurons spiking behavior in vitro, and can be used to develop intelligent molecular processing systems in vivo.

## Introduction

Biomolecular computation refers to the study of exploiting biological macromolecules to implement relatively standard methods of computation, including molecular computing [1]–[3], membrane computing [4],[5], storage media using bacteria rhodopsin [6],[7] and biologically altered cells that do rudimentary operations within the paradigm of traditional computation, etc. In recent years, computing with programming DNA molecular has become a hot research topic and lot of work have contributed to this field, such as helical molecular programming [8], tabletop molecular communications with chemical signals [9], molecular computing methods to improve the accuracy of insertion site analysis in tumors [10], DNA self-assembly for computation [11],[12], DNA strand displacement (DSD) technology [13],[14].

DNA strand displacement technology has been proposed as an isothermal, in vitro DNA amplification technique in [13]. The technique is highly selective to the recognition of sequence [15], and has been used in detection of gene signals as a second-generation DNA probe system with the help of some DNA nanotechnology, see e.g. in [16]–[22]. DNA strand displacement is an isothermal and enzyme free technique, see e.g. [13],[16], and it can be potentially applied as an intelligent molecular systems in vivo for DNA signal detection and processing. Some bio-molecular signal processing systems have been developed with using DNA strand displacement technology, such as enzyme-free nucleic acid logic circuits [23], genetic programming and evolvable molecular machines [24], performing logic computation of Hopfield network auto-associative memory with DNA strands [25], kinetically controlled self-assembly of DNA oligomers [26].

In bio-molecular signal processing systems proposed in [23]–[26], single-stranded DNA molecules are generally used as input and output signals; all the strands needed for the computation are mixed together, and then the computation proceeds according to the design of DNA sequences without further intervention. The energy for this procedure is provided by the Watson-Crick complementary mechanism of DNA structures themselves, so whole system can run in basic wet labs. Note that all DNA strands can interact with each other automatically according to programmed logic with DNA strand displacement technology, that is, each DNA strand performs its own reactions independently with logically related strands respectively. The DNA strands cannot “cross talk” with other unrelated strands restricted by sequence design.

A molecular system can be seemed as a functioning unit with DNA strands representing particular partial biological functions, where information is encoded and stored in form of DNA strands, and each DNA strand performs its own reactions independently to achieve particular biological function. The output DNA strands in certain reaction can be taken as input of another DNA strand reactions, which in some sense can process information like functioning unit performing chain reactions.

## Results

In this work, we present a novel method to achieve logic gate computation with DNA strand displacement technology. In general, group of “DNA neurons” are designed, and by cascading organization of the “DNA neurons” some intelligent molecular systems for logic gate computation are achieved.

In “DNA neurons”, we use a “smart” way that neurons encoding information by means of accumulation of spikes to encode information with “accumulation” of volumes of the input signal DNA molecules. Specifically, we use a amount of mol of pre-designed DNA strand as a basic information unit, and then encode information by different amounts of mols of basic DNA molecules. As well, some logically separated group of DNA strands, named “DNA neuron”, are designed for a particular reaction, which can perform its own function in a “separated region” (without interactions with other “DNA neurons”). The communication of “DNA neurons” with each other is as follows: one “DNA neuron” performs its own reactions to generate particular output DNA strands, and the other “DNA neuron” starts its reaction only when it senses the generated output DNA strands.

With “DNA neurons”, we construct intelligent molecular systems for logic gates computation, including AND, OR, XOR gates. The systems perform logic computation with each “DNA neuron” detecting and processing DNA signals automatically until the computation result (which can be reported by “reporter DNA strand”) is obtained. To show the validity of our method, we firstly use visual DSD from [27] to do simulations of the computations in the systems for AND, OR, XOR logic gates, and then experiments are performed. Experimental results show that the systems with “DNA neurons” can work correctly and efficiently for performing logic gate computations, including AND, OR, and XOR logic gates.

## Methods and Materials

### Method

In general, “DNA neurons” can receive different combination of ssDNA strands as input spikes, and then release corresponding ssDNA strands as output signals. These output signals could be accepted by other DNA neurons as input spikes. This property makes it possible to cascade these DNA neurons systematically to build more complicated circuits. The XOR logic is such a creation that is constructed by organizing two AND logics in an artful way. The constructed XOR logic has an excellent attribute that the output is determined with given input spikes, which will be explained in details later.

**Figure 1:**
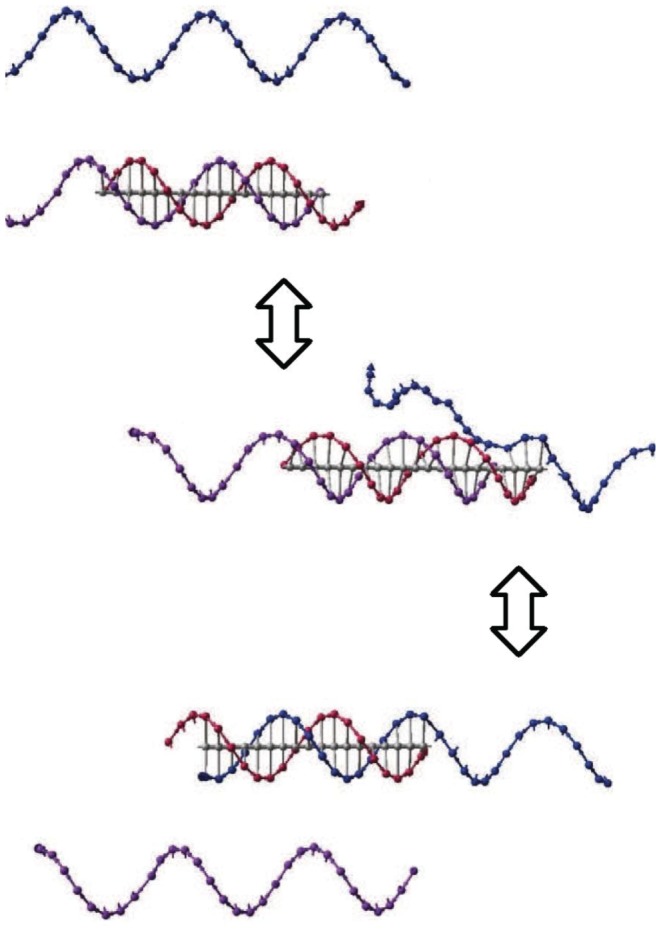
Thresholding reaction of a DNA neuron.

**Figure 2:**
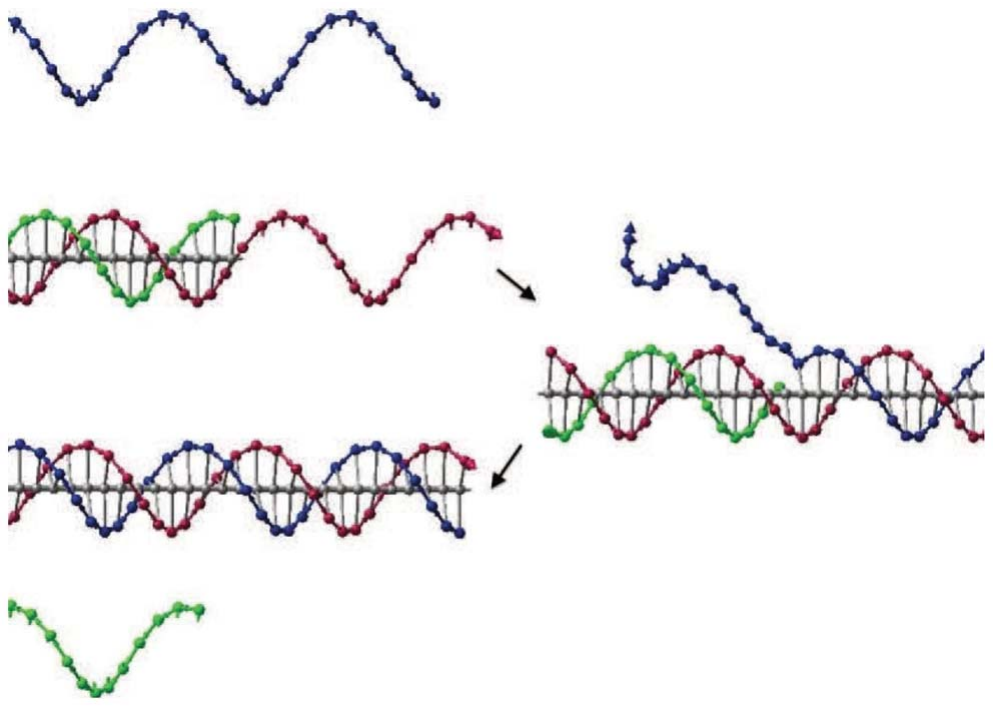
Irreversible DNA strand displacement.

There are two kinds of DNA strand displacement strategies applied in this work: one is reversible strand displacement (shown in Figure 1), the other one is irreversible strand displacement (shown in Figure 2). For reversible strand displacement reaction, the input strand (blue strand in Figure 1) hybridizes with a gate complex (red and purple complex in Figure 1) through the exposed toehold, and then the branch migrates through recognition domain occurs and finally the previous strand (purple strand in Figure 1) will desquamate as an output signals of a DNA neuron. Note that there is the same uncovered toehold on the opposite side of the gate base strand (read strand in Figure 1), which makes this reaction reversible. In irreversible reaction, the input strand (blue strand in Figure 2) hybridizes with a threshold complex (red and green complex in Figure 2) via an extended toehold, and the original branch (green strand in Figure 2) bound to the threshold is replaced, and this reaction is not reversible since there is no uncovered toehold left.

**Figure 3:**
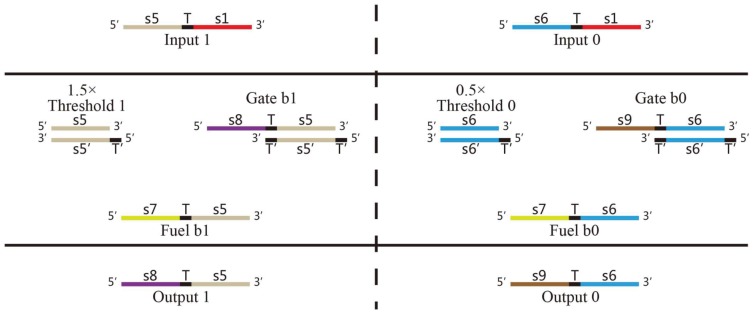
Abstract diagrams of implementation of a DNA neuron with AND logic.

Bistable DNA neurons with two stable output states are designed based on the two basic DNA strand displacement reactions above. It is shown how does the DNA neuron execute AND logic in Figure 3. The bistable DNA neuron can be divided in two parts. It is given in the left of Figure 3 the positive part which receives and releases the positive signal “1” and in the left of Figure 3 is the negative part corresponding with the negative neuron signal “0”. Each part consists of input, threshold, gate, fuel and output strands. The input strands is added in a form of accumulation of impulses, i.e., double amounts of input strand “1” added to represents the input of “11”; one time amount of input strand “0” and one time amount of input strand “1” represent the input signal of “01”; and double amounts of input strand “0” represent the input signal of “00”. It is clear that rather than hybridizing with the gate, the input strand signals prefer to hybrid with the threshold since this reaction is irreversible. The threshold value in the positive part is 1.5, thus only when the input strand is “11”, an output signal strand in the positive part can be expected. The threshold value in the negative part, similarly, is set to be 0.5, thus output signal strand in the negative part can be generated only when the input signals are “10”, “01” and “00”. In this way, only if the input is “11”, the Output 1 strand could be detected; otherwise Output 0 strand will be detected. Inversely, if Output 1 strand is regarded as negative output signal 0, and Output 0 strand is regarded as output of positive signal 1, then AND logic can perform logic computation of OR logic gate.

**Figure 4:**
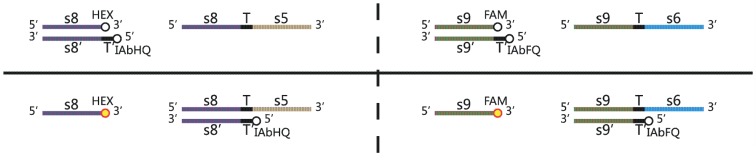
HEX and FAM fluorescent probes to detect the output spike “1/0” of DNA Neuron.

In order to detect the strand representing positive output signal “1” and strand representing negative signal strand “0”, two kinds of fluorescent probes, namely reporter strands, are designed as shown in Figure 4. The reporter strands for output strand signals “1” and “0” (strand 8 and strand 9 in Figure 4) are labeled by fluorophores (HEX and FAM in Figure 4) and quenchers (IAbHQ and IAbFQ in Figure 4) occurring fluorescence quenching. When the output strand representing signal “1” or “0” releases from DNA neuron, it will trigger the correspondence reporter and a irreversible DNA strand displacement take place. In this way, the two radicals will be separated at last and the fluorescence signal representing “1” or “0” can be monitored with a real-time PCR Machine.

**Figure 5:**
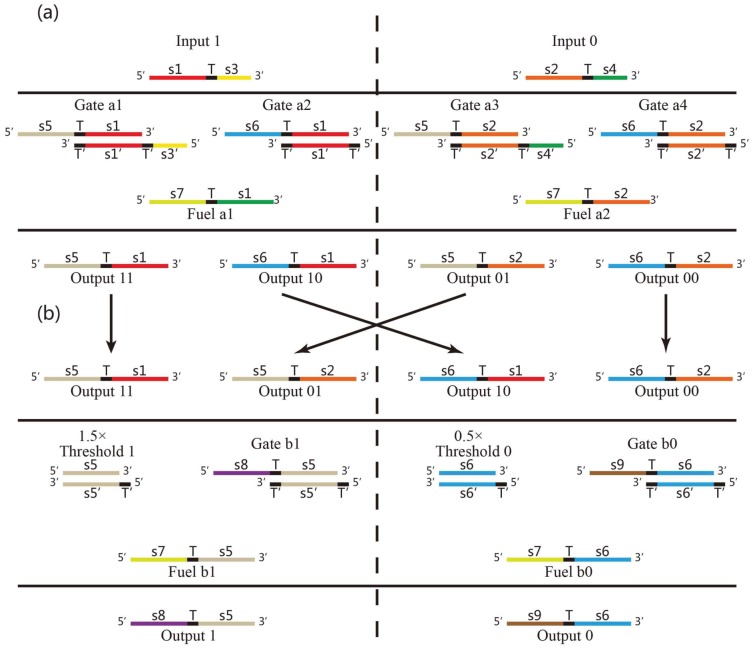
Abstract diagrams of two DNA neurons executing XOR logic.

The XOR logic gate is constructed by cascading two DNA neurons executing AND logics in an artful way, which consists of two parts, XOR (a) and XOR (b), which is respectively illustrated in Figure 5 (a) and (b). In each part, it can generate two outputs: a positive output (Output signal strand “11” or “01”), and a negative output (Output signal strand “10” or “00”), the signal strands can be transferred to the DNA neuron in XOR (b) as input signals. It is noted that only input signal strands with doubled amount would generate negative output. Specifically, by receiving input signal “11”, XOR (a) will release one time amount of positive output (Output strand “11”) and one time amount of negative output (Output strand “10”), while by receiving the input signal “10”, it will release double amount of positive output (Output strand “11” and “01”). The output signals of XOR (a) can be taken as input signal of XOR (b). If XOR (b) receives double amounts of positive input signals, it leads to release Output strand “1”; otherwise, it output negative signal by releasing Output strand “0”. With the explanation above, the system performing computation of XOR logic gate is constructed with DNA neurons. To make the computing process of DNA neurons for XOR logic clear, the following cases are discussed, where by 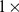 we denote the basic amount of mol as a basic information unit.

- We add 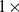 strand Input 0 and 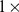 strand Input 1 into the sample to represent the input signal being “01”, and then the Gate a1 and Gate a3 reaction can occur respectively. Eventually, the positive part will output positive signal (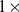 strand Output strand “11”) and the negative part will output positive signal (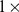 Output strand “01”). The outputs can be sensed as input signals of XOR (b). In XOR (b), if the concentration of the two positive input strands exceeds the threshold value 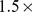, and it outputs positive signal “1” consequently.
- We add 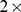 strand Input 1 into the sample to represent the input signal being “11”. The Gate a1 and Gate a2 reactions occur successively in the positive part. Ultimately the positive part will output positive signal (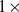 Output strand “11”) and negative spike (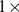 Output strand “10”). The output strands will enter into XOR (b) as input signals, in which, the concentration of the negative signal will exceeds the threshold value 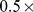, and it outputs negative signal strand “0” consequently.
- As for input signal “00”, the XOR (a) release 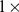 Output strand “01” and 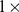 Output strand “00”. XOR (b) can sense these strands as input signal “10” and release negative signal “0”.

The design of DNA neuron executing AND, OR, XOR logic gates with input based on amount variation is rational and has potential to be applied to construct circuit with more complexity.

### Simulation

Based on the DSD mechanism analysis above, Visual DSD from [27] (a software package that could visualize the species and the reactions of the process of DNA strand displacement at the domain level then generate stochastic or deterministic simulations) is introduced as a tool of kinetics simulation to verify the feasibility of the bistable DNA neuron design. The models of AND logic and XOR logic gates are both programmed with Visual DSD, and for each model three types of input signals, “00”, “01” and “11' are tested with expected output. The strands used in the kinetics simulations are the same with the wet experiment that followed.

**Figure 6:**
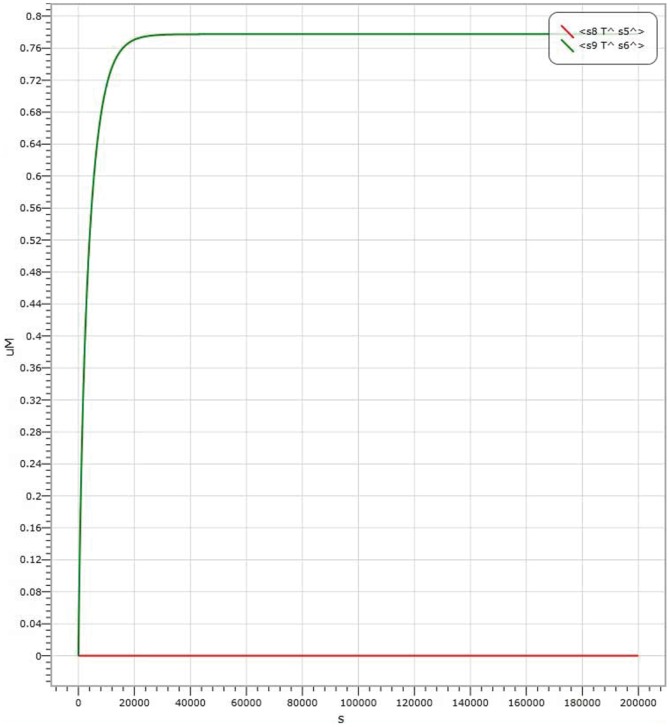
Visual DSD simulations of AND logic gate with input signal “00”.

**Figure 7:**
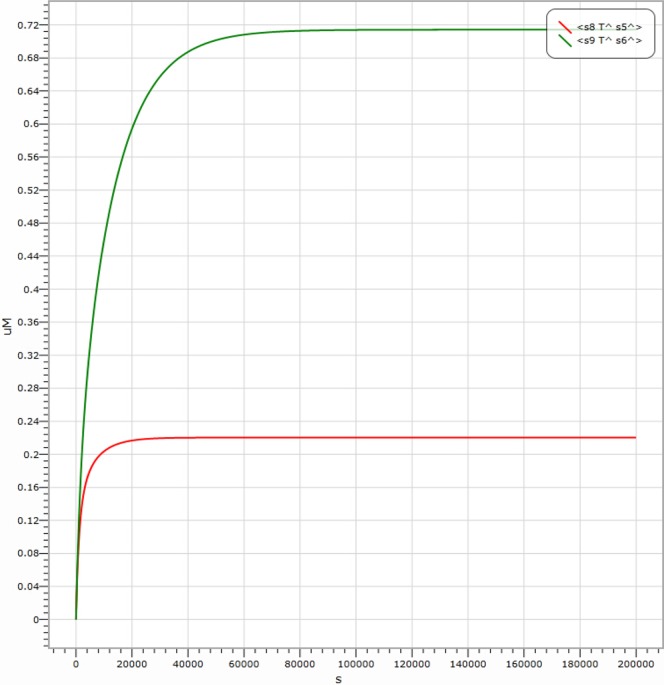
Visual DSD simulations of AND logic gate with input signal “01”.

**Figure 8:**
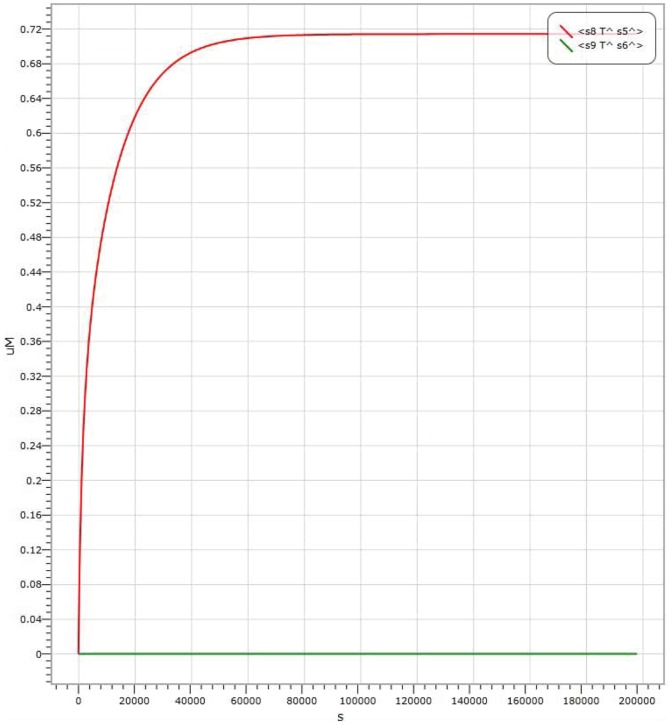
Visual DSD simulations of AND logic gate with input signal “11”.

In AND logic gate, shown in Figure 4, the amount of Threshold 1 is 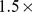; that of Threshold is 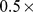; that of Gate b1 and that of Gate b0 are both 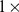, while Threshold of Fuel b1 and Fuel b0 are both 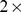. The results of kinetics simulations are shown in Figures 6, 7 and 8, where the red curve denotes the amount of molecule P15 (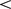 s8 T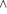 s5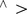) representing the output of signal “1” and the green curve denotes the amount of molecule P20 (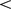 s9 T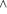 s6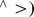 representing the output signal of “0”. It is shown in Figure 6 the simulation result with the input signal of “00” (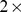 Input strand 0, representing by molecule labelled with P7); the simulation result with the input signal of “01” is indicated in Figure 7, where the output is 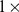 Input strand 0 and 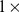 Input 1 strand representing by molecule labelled with P1); the simulation result with the input signal of “11” is shown in Figure 8. It is worth to point out that in Figures 6, 7 and 8, there are large margins to distinguish the right result from the wrong. It means the molecular system for performing computation of ADD logic gate is with high confidence level.

**Figure 9:**
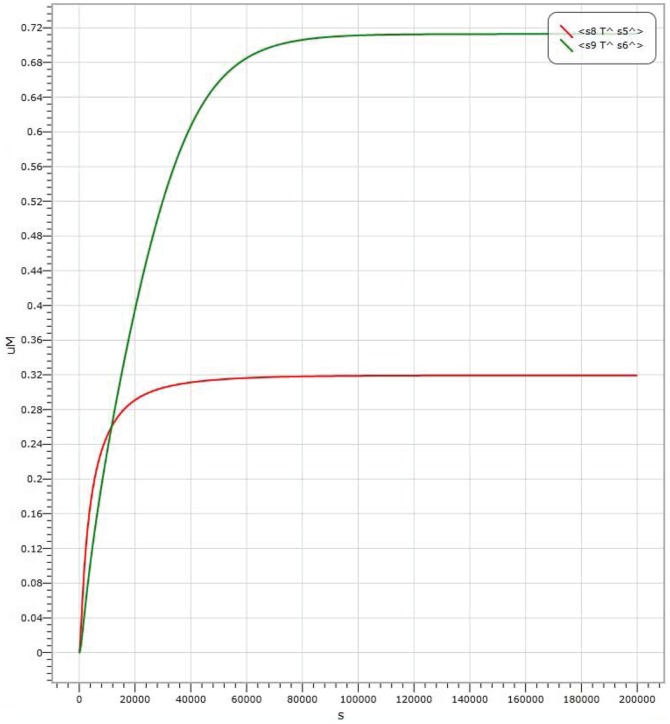
Visual DSD simulations of XOR logic gate with input signal “00”.

**Figure 10:**
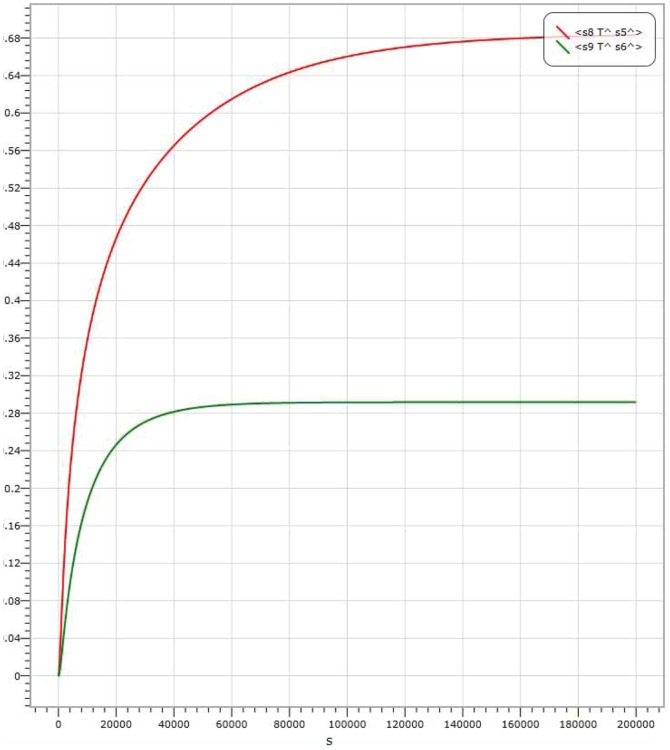
Visual DSD simulations of XOR logic gate with input signal “01”.

**Figure 11:**
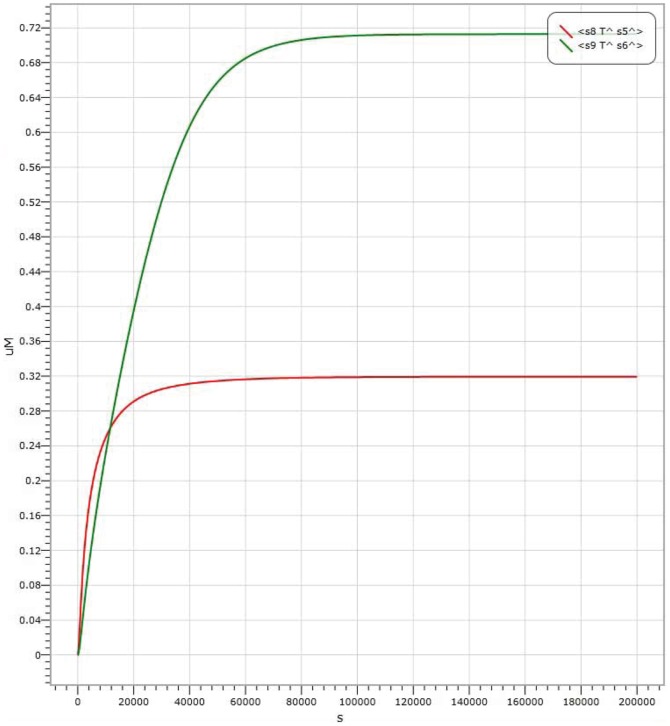
Visual DSD simulations of XOR logic gate with input signal “11”.

The simulation results of XOR logic gate are shown in Figures 9, 10 and 11. In the figures, red curves represent the amount of molecules representing positive signal “1” and green curves represent the amount of molecules representing negative signal “0”. The detailed amounts of involved DNA molecules go as shown in Table 1.

**Table 1:** The detailed amounts of involved DNA molecules in XOR logic gate.

Name	Gate a1	Gate a2	Gate a3	Gate a4	Fuel a1	Fuel a2
Amount	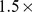	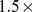	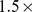	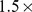	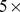	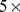
Name	**Threshold 1**	**Gate b1**	**Threshold 0**	**Gate b0**	**Fuel b1**	**Fuel b0**
Amount	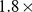	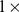	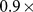	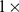	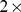	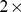

## Experiments

### Materials

#### DNA Oligodeoxynucleotide Strands

**Figure 12:**
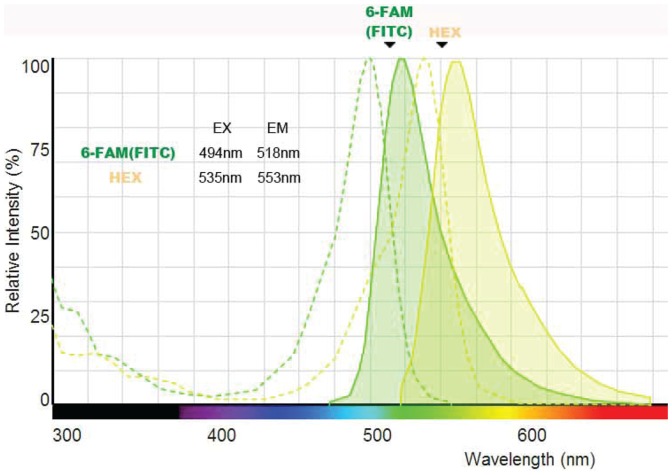
Fluorescence spectra of 6-FAM(FITC) and HEX.

All the DNA strands used in the experiments are purchased and from Sangon Biotech (Shanghai, China) Co., Ltd. Most of them are with PAGE purification expect fluorescent ones. The DNA strands, purified by HPLC, are 3′-labeled respectively with fluorophore 6-FAM (FITC), HEX and 5′-labeled with other corresponding quenchers. Fluorescence spectra of two fluorophores are shown in Figure 12 (Fluorescence SpectraViewer from Thermo Fisher Scientific Inc). It confirms little interference between two sets of wavelengths of excitation (dotted curves) and emission (solid curves).

#### Reagents and Equipment

All the mixtures are dissolved with ultrapure water. 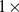 TAE/Mg2+ buffer consists of 40 mM Tris (pH 7.6), 2 mM EDTA, 20 mM acetic acid and 35 mM magnesium acetate. The whole reactions are occurred in the real-time PCR from Xi'An TianLong Science and Technology Co., Ltd.

#### Oligonucleotide Sequences

We design 26 different DNA strands with reusable domains for both AND logic and XOR logic of “DNA neurons”. Sequences of the strands form P1 to P26 and reusable domains from s1 to s10 are listed in Tables 2 and 3. Component samples of “DNA neurons” constructed from T1 to T16 are shown in Table 4 and compositions of fluorescent probes highlight in Table 5.

**Table 2:** All Oligonucleotides for “DNA neurons”.

ID	Domains	Sequence (5′-3′)
P1	s1+T+s3	CACCCTAAAATCTCATCTCACATAACA
P2	s5+T+s1	CATCCATTCCACTCATCTCACCCTAAAATCTCA
P3	s3′+T′+s1′+T′	TGTTATGTGAGATGAGATTTTAGGGTGAGA
P4	s6+T+s1	CACCACCAAACTTCATCTCACCCTAAAATCTCA
P5	T′+s1′+T′	AGATGAGATTTTAGGGTGAGA
P6	s7+T+s1	CAACATATCAATTCATCTCACCCTAAAATCTCA
P7	s2+T+s4	CATAACACAATCACATCTCAAAACAAA
P8	s5+T+s2	CATCCATTCCACTCATCTCATAACACAATCACA
P9	s4′+T′+s2′+T′	TTTGTTTTGAGATGTGATTGTGTTATGAGA
P10	s6+T+s2	CACCACCAAACTTCATCTCATAACACAATCACA
P11	T′+s2′+T′	AGATGTGATTGTGTTATGAGA
P12	s7+T+s2	CAACATATCAATTCATCTCATAACACAATCACA
P13	s5	CATCCATTCCACTCA
P14	T′+s5′	AGATGAGTGGAATGGATG
P15	s8+T+s5	CACCATCAAATAACATCTCATCCATTCCACTCA
P16	T′+s5′+T′	AGATGAGTGGAATGGATGAGA
P17	s7+T+s5	CAACATATCAATTCATCTCATCCATTCCACTCA
P18	s6	CACCACCAAACTTCA
P19	T′+s6′	AGATGAAGTTTGGTGGTG
P20	s9+T+s6	CACTAACATACAACATCTCACCACCAAACTTCA
P21	T′+s6′+T′	AGATGAAGTTTGGTGGTGAGA
P22	s7+T+s6	CAACATATCAATTCATCTCACCACCAAACTTCA
P23	PC+T′+s8′	TGAGATGTTATTTGATGGTG
P24	s8	CACCATCAAATAACA
P25	PC+T′+s9′	TGAGATGTTGTATGTTAGTG
P26	s9	CACTAACATACAACA

**Table 3:** Domains of “DNA neurons”.

Name	Sequence	Length
T	TCT	3
PC	TG	2
s1	CACCCTAAAATCTCA	15
s2	CATAACACAATCACA	15
s3	CACATAACA	9
s4	CAAAACAAA	9
s5	CATCCATTCCACTCA	15
s6	CACCACCAAACTTCA	15
s7	CAACATATCAATTCA	15
s8	CACCATCAAATAACA	15
s9	CACTAACATACAACA	15

**Table 4:** Components samples.

Sample ID	Formation	Function
T1	P1	Input 1
T2	P2 P3	Gate a1
T3	P4 P5	Gate a2
T4	P6	Fuel a1
T5	P7	Input 0
T6	P8 P9	Gate a3
T7	P10 P11	Gate a4
T8	P12	Fuel a2
T9	P13 P14	Threshold 1
T10	P15 P16	Gate b1
T11	P17	Fuel b1
T12	P18 P19	Threshold 0
T13	P20 P21	Gate b0
T14	P22	Fuel b0
T15	P23 P24	Reporter 1
T16	P25 P26	Reporter 0

**Table 5:** Probes with Fluorophores.

Strand	Probe	Sequences (5′-3′)
P23	Reporter 1	TGAGATGTTATTTGATGGTG/3HEX/
P24	Reporter 1	/5IAbHQ/CACCATCAAATAACA
P25	Reporter 0	TGAGATGTTGTATGTTAGTG/36-FAM/
P26	Reporter 0	/5IAbFQ/CACTAACATACAACA

### Experimental Process

The standard concentration, 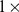 DNA strands, is 20 nM.

1. To form a double strand, two specified single DNA strands, named strand 1 and strand 2, are added into 20lof solution which contains 4 *µ*M strand a, 4 *µ* M strand b, 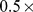 TAE/Mg2+buffer. There are ten different solutions included double strands that are formed by strand a and strand b. They are corresponding respectively to P2 and P3 for T2, P4 and P5 for T3, P8 and P9 for T6, P10 and P11 for T7, P13 and P14 for T9, P15 and P16 for T10, P18 and P19 for T12, P20 and P21 for T13, P23 and P24 for T15, P25 and P26 for T16. All the solutions are incubated at 95 °C for 3 minutes and then cooled down to 4 °C for 16 hours.
2. Mix with 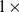 P15, 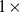 P20, 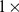 T15, 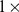 T16 and 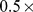 TAE/Mg2+ buffer into the standard solution. Incubate at 25 °C for more than 8 hours.
3. 100 *µ*l mixture of a DNA neuron AND logic includes 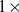 T10, 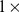 T13, 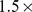 T9, 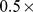 T12, 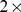 P17, 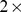 P22, 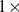 T15, 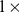 T16 and 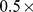 TAE/Mg2+ buffer. Each of the three solutions has the different concentration of input strands. The mixture of the input signal “11” contains 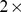 P1; the mixture of the input “01/10” contains 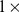 P1, 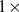 P7; the mixture of the input “00” contains 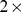 P7. All the mixtures incubate in the qRT-PCR at 25 °C for 8 hours.
4. A DNA neuron for XOR logic gate is 100 *µ*l mixture of XOR (a) and XOR (b). XOR (a) solution includes 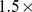 T2, 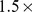 T3, 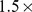 T6, 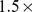 T7, 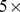 P6, 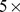 P12 and 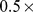 TAE/Mg2+buffer. XOR (b) includes 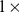 T10, 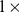 T13, 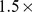 T9, 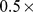 T12, 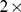 P17, 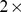 P22, 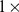 T15, 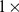 T16 and 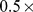 TAE/Mg2+buffer. Then put two solutions XOR (a) and XOR (b) together as XOR logic mixture.

## Experimental Results

Designed for the experiment, the solutions containing specific DNA strands deploy “DNA neurons”. Two fluorescent probes T15 and T16 compose reporter “1” and reporter “0” to detect the output signal of “DNA neurons”. The standard solution is for measures of fluorescent detection of two channels. P2 and P4 represent input signals for “DNA neurons”; P1 corresponds to positive signal “1” and P7 represents negative signal “0” of AND logic gate shown in Figure 4.

**Figure 13:**
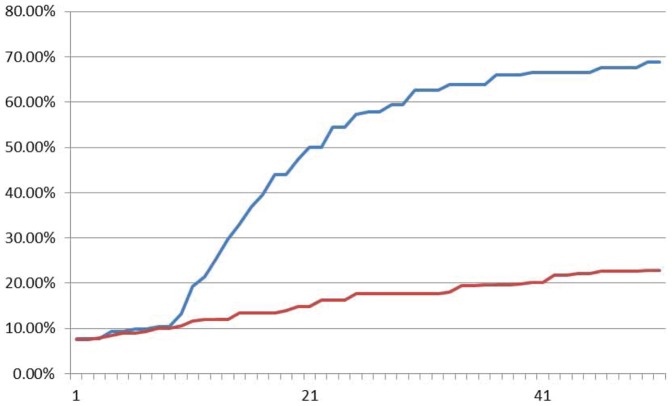
Fluoroscence data of DNA Neurons execute AND logic with input “00”. The X-axis is cycling time of Real-time PCR, the time span of each cycle is 10 minutes, temperature of each cycle keeps in 24–25°C. The Y-axis is relative intensity of HEX (red curve) and FAM (blue curve).

**Figure 14:**
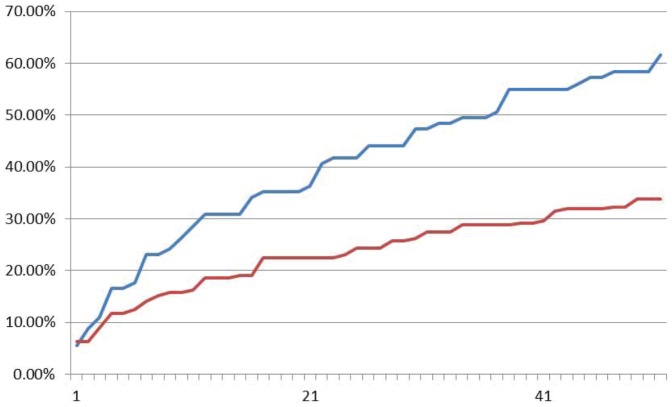
Fluoroscence data of DNA Neurons execute AND logic with input “01”. The X-axis is cycling time of Real-time PCR, the time span of each cycle is 10 minutes, temperature of each cycle keeps in 24–25°C. The Y-axis is relative intensity of HEX (red curve) and FAM (blue curve).

**Figure 15:**
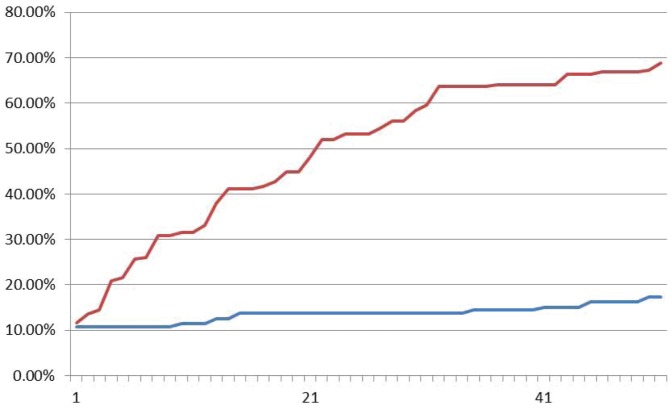
Fluoroscence data of DNA Neurons execute AND logic with input “11”. The X-axis is cycling time of Real-time PCR, the time span of each cycle is 10 minutes, temperature of each cycle keeps in 24–25°C. The Y-axis is relative intensity of HEX (red curve) and FAM (blue curve).

Fluoroscence data of DNA neurons execute AND logic are shown in Figures 13, 14 and 15 with input signals “00”, “01” and “11”, respectively. As it is shown in Figure 13, the relative intensity of FAM signal that represents negative output signal “0” increases gradually from about 10% up to about 70%, while the HEX signal that represents positive output signal “1” keeps at about 10% to 20% in the sample with input signal “00”. It means the output signal is “0”. In Figure 15, the plot of input signal “11” displays that HEX signal intensity of positive signal “1” increases gradually from about 10% up to about 70%, while FAM signal intensity keeps below 20%. In Figure 14, the situation is illustrated in the sample with input “01/10”. The intensities of both HEX and FAM signals has the parallel trace and the similar growth during the reaction, while the FAM signal growth two times faster than HEX signal. It is shown that all the correct strands can be detected from the AND logic gate.

In the solution of XOR logic indicated in Figure 5, the calculation in XOR (b) is the same with he AND logic gate. The differences are the changes of the input signals and the addition of XOR (a) including Gate a1, a2, a3, a4, Fuel a1 and a2. By mixing XOR (a) and XOR (b), the molecular system are cascaded as XOR logic gate.

**Figure 16:**
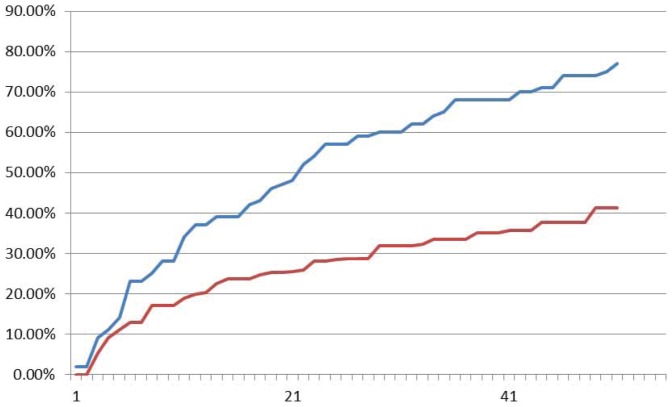
Fluoroscence data of DNA Neurons execute XOR logic with input “00”. The X-axis is cycling time of Real-time PCR, the time span of each cycle is 10 minutes, temperature of each cycle keeps in 24–25°C. The Y-axis is relative intensity of HEX (red curve) and FAM (blue curve).

**Figure 17:**
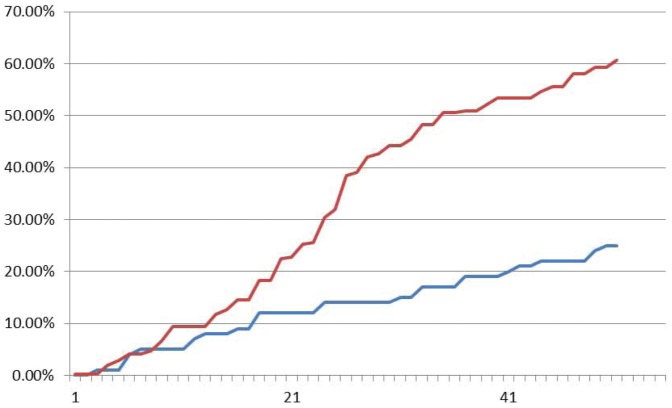
Fluoroscence data of DNA Neurons execute XOR logic with input “01”. The X-axis is cycling time of Real-time PCR, the time span of each cycle is 10 minutes, temperature of each cycle keeps in 24–25°C. The Y-axis is relative intensity of HEX (red curve) and FAM (blue curve).

**Figure 18:**
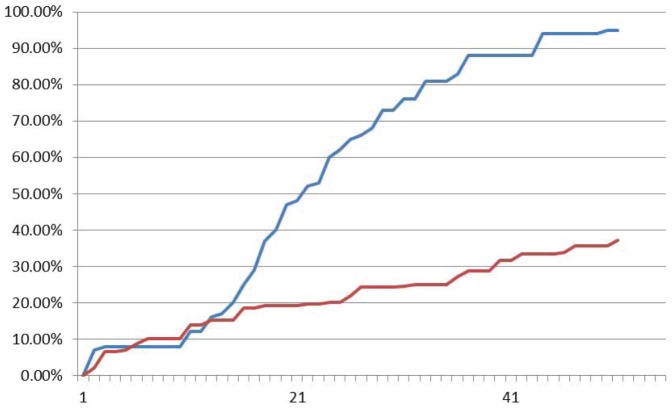
Fluoroscence data of DNA Neurons execute XOR logic with input “11”. The X-axis is cycling time of Real-time PCR, the time span of each cycle is 10 minutes, temperature of each cycle keeps in 24–25°C. The Y-axis is relative intensity of HEX (red curve) and FAM (blue curve).

Experimental results by fluorescence data of XOR logic gate are shown in Figures 16, 17 and 18 with input signals “00”, “01/10” and “11” respectively. It demonstrates in Figure 16 that fluorescent signal intensity of FAM (representing output signal “0”) increases gradually from zero to about 80% and that of HEX (representing output signal “1”) keeps below 40% in the sample with input signal “00”. A similar case happens in the sample with input signal “11” shown in Figure 18. The fluorescent signal intensity of FAM (representing output signal “0”) keeps below 40%, but signal intensity HEX (representing output signal “1”) increases gradually from zero to about 80%. In the sample with input signal “01/10”, it performs that intensity of HEX increases gradually from about zero up to above 60% and that of FAM keeps below 30% see Figure 17. All the plots clarify that it takes out correct strands through the XOR logic.
